# Crystal structure of 2-fluoro-*N*-(1,3-thia­zol-2-yl)benzamide

**DOI:** 10.1107/S2056989015019192

**Published:** 2015-10-24

**Authors:** Rodolfo Moreno-Fuquen, Juan C. Castillo, Diana Becerra, Hernando Camargo, José A. Henao

**Affiliations:** a Departamento de Química – Facultad de Ciencias Naturales y Exactas, Universidad del Valle, Apartado 25360, Santiago de Cali, Colombia; b Departamento de Química, Universidad de los Andes, Carrera 1 No 18A12, Bogotá, Colombia; c Facultad de Química Ambiental, Universidad Santo Tomás, Campus Universitario Floridablanca, Santander, Colombia; d Escuela de Química, Facultad de Ciencias, Universidad Industrial de Santander, Apartado 678, Bucaramanga, Colombia

**Keywords:** crystal structure, thia­zole derivatives, cancer cell-growth inhibitors, carboxamides, 1,3-thia­zole, benzamide, hydrogen bonding

## Abstract

In the title compound, C_10_H_7_FN_2_OS, the mean plane of the central amide fragment (r.m.s. deviation = 0.048 Å) makes dihedral angles of 35.28 (8) and 10.14 (12)° with those of the fluoro­benzene and thia­zole rings, respectively. The thia­zole S and amide O atoms lie to the same side of the mol­ecule. In the crystal, pairs of N—H⋯N hydrogen bonds connect the mol­ecules into inversion dimers with *R*_2_^2^(8) motifs, and weak C—H⋯O inter­actions connect the mol­ecules into *C*(6) [001] chains. Together, the N—H⋯N and C—H⋯O hydrogen bonds generate (100) sheets.

## Related literature   

For thia­zole derivatives as inhibitors for cancer cell growth, see: Schade *et al.* (2008 ▸). For carboxamides with synthetic and biological inter­est, see: Moreno-Fuquen *et al.* (2014*a* ▸,*b* ▸). For related structures, see: Zonouzi *et al.* (2009 ▸); Saeed *et al.* (2010 ▸).
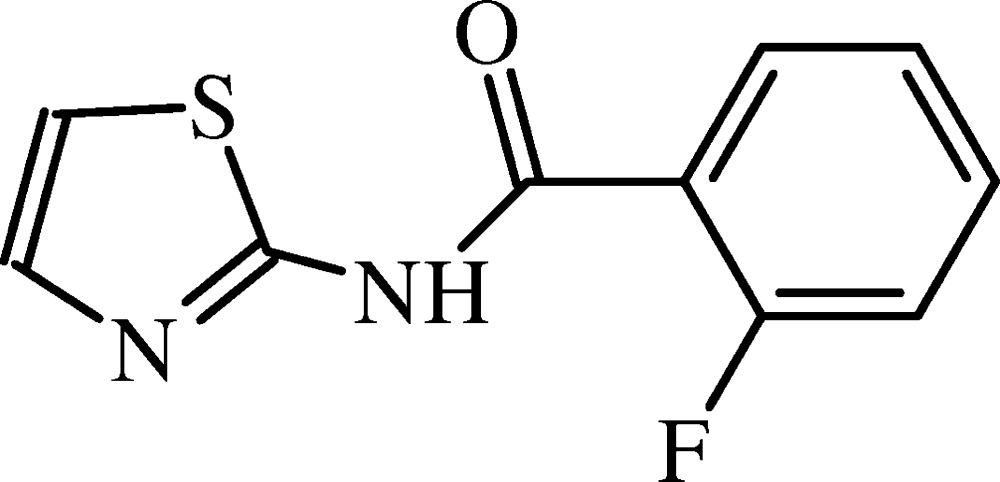


## Experimental   

### Crystal data  

C_10_H_7_FN_2_OS
*M* *_r_* = 222.24
Monoclinic, 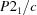

*a* = 12.2171 (8) Å
*b* = 5.0741 (3) Å
*c* = 15.7078 (10) Å
β = 98.820 (6)°
*V* = 962.22 (11) Å^3^
*Z* = 4
Mo *K*α radiation
μ = 0.32 mm^−1^
*T* = 295 K
0.40 × 0.17 × 0.08 mm

### Data collection  

Rigaku Pilatus 200K diffractometer
Absorption correction: multi-scan *CrystalClear*; Rigaku, 2008 ▸ *T*_min_ = 0.701, *T*_max_ = 1.000
8722 measured reflections
2169 independent reflections
1556 reflections with *I* > 2σ(*I*)
*R*_int_ = 0.060

### Refinement  

*R*[*F* ^2^ > 2σ(*F* ^2^)] = 0.046
*wR*(*F* ^2^) = 0.098
*S* = 0.89
2169 reflections
136 parameters
H-atom parameters constrained
Δρ_max_ = 0.22 e Å^−3^
Δρ_min_ = −0.30 e Å^−3^

### 

Data collection: *CrystalClear* (Rigaku, 2008 ▸); cell refinement: *CrystalClear*; data reduction: *CrystalClear*; program(s) used to solve structure: *SHELXS2014* (Sheldrick, 2008 ▸); program(s) used to refine structure: *SHELXL2014* (Sheldrick, 2015 ▸); molecular graphics: *ORTEP-3 for Windows* (Farrugia, 2012 ▸) and *Mercury* (Macrae *et al.*, 2006 ▸); software used to prepare material for publication: *WinGX* (Farrugia, 2012 ▸).

## Supplementary Material

Crystal structure: contains datablock(s) I, global. DOI: 10.1107/S2056989015019192/hb7520sup1.cif

Structure factors: contains datablock(s) I. DOI: 10.1107/S2056989015019192/hb7520Isup2.hkl

Supporting information file. DOI: 10.1107/S2056989015019192/hb7520Isup3.cml

**Fig. 1.**
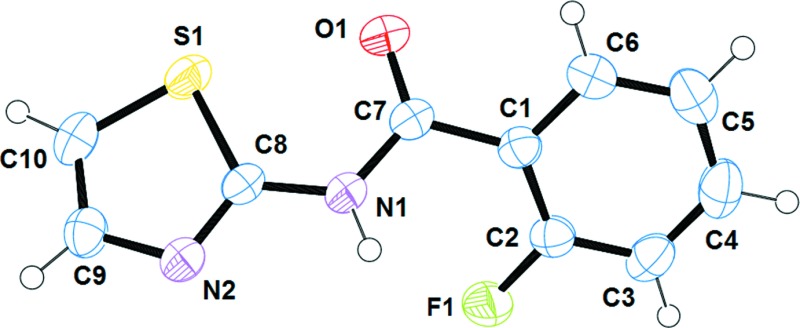
The molecular structure of (I) with displacement ellipsoids drawn at the 50% probability level.

**Fig. 2.**
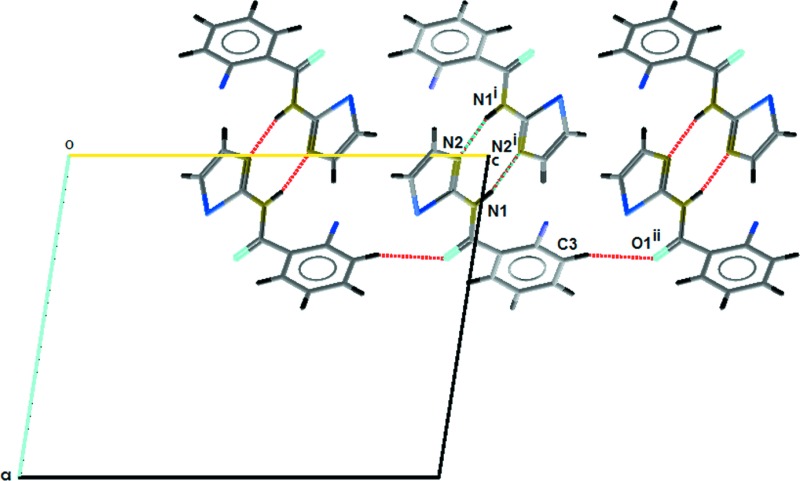
Part of the crystal structure of (I), showing the formation of hydrogen-bonded C(13) chains parallel to [311] [Symmetry code: (i) -x - 1/2, y - 1/2, -z + 1/2].

. DOI: 10.1107/S2056989015019192/hb7520fig1.tif
The mol­ecular structure of (I) with displacement ellipsoids drawn at the 50% probability level.

x y z . DOI: 10.1107/S2056989015019192/hb7520fig2.tif
Part of the crystal structure of (I), showing the formation of hydrogen-bonded C(13) chains parallel to [31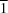] [Symmetry code: (i) −*x* − 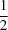, *y* − 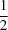, −*z* + 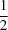].

CCDC reference: 1430605

Additional supporting information:  crystallographic information; 3D view; checkCIF report

## Figures and Tables

**Table 1:** Hydrogen-bond geometry (, )

*D*H*A*	*D*H	H*A*	*D* *A*	*D*H*A*
N1H1N2^i^	0.86	2.11	2.944(2)	165
C3H3O1^ii^	0.93	2.62	3.474(2)	153

Symmetry codes: (i) 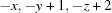; (ii) 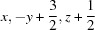.

## supplementary crystallographic information

### S1. Comment

Continuing with our current studies on the synthesis of new N-heterocyclic carboxamide derivatives of synthetic and biological interest (Moreno-Fuquen *et al.*, 2014a, Moreno-Fuquen *et al.*, 2014b), the title compound 2-fluoro-N-(thiazol-2-yl)benzamide (I) was obtained by direct reaction of 2-fluorobenzoyl chloride and 2-aminothiazole in the presence of triethylamine as base under mild conditions. Structures of similar molecules were compared with (I), i.e. N-(1,3-thiazol-2-yl)benzamide (Zonouzi *et al.*, 2009) and 2,4-dichloro-N-(1,3-thiazol-2-yl)benzamide (Saeed *et al.*, 2010). The molecular structure of (I) is shown in Fig. 1. The central amide moiety, C8-N1-C7(-O1)-C1, is essentially planar (r.m.s. deviation for all non-H atoms = 0.048 Å) and it forms dihedral angles of 35.28 (8)° with the C1-C6 ring and 10.14 (12)° with the thiazole ring. The C=O bond is *anti* to the o-F1 substituent in the aromatic ring. The N-H and C=O bonds in the central amide group are also *anti* to each other. Comparing (I) with the two aforementioned similar structures, reveals that significant differences in bond lengths and bond angles are not observed. In the crystal structure, dimer formation is observed. Molecules of (I) are linked by hydrogen bonding of moderate strength. The N-H group of the central amide moiety, in the molecule at (x,y,z) acts as hydrogen bond donor to N2 atom of the thiazole molecule at (-x,-y+1,-z+2), (see Table 1). In turn these dimers are connected by weak hydrogen bonds: The C-H group in the molecule at (x,y,z) acts as hydrogen bond donor to carbonyl O1 atom in the molecule at (x,-y+3/2,z+1/2), forming chains C(6) of molecules along [001], see Fig. 2.

### S2. Experimental

2-Fluorobenzoyl chloride (143 µl, 1.2 mmol) was added dropwise to a solution of 2-aminothiazole (100 mg, 1.0 mmol) and triethylamine (278 µl, 2.0 mmol) in dichloromethane (3.0 mL). The mixture was stirred at room temperature for 4 h until the starting amine was not longer detected by thin-layer chromatography. After solvent was removed under reduced pressure, the resulting solid was dissolved in H_2_O (3.0 ml) and extracted with EtOAc (2 × 3.0 ml). The combined organic layers were dried with MgSO_4_ anhydrous and the solvent was removed under reduced pressure to afford the pure amide product. Colourless plates of (I) were grown by slow evaporation, at room temperature and in air, from a solution in methanol [61% yield, m.p. 443 (1) K].

### S3. Refinement

All H-atoms were located in difference Fourier maps and were positioned geometrically [C—H = 0.93 Å for aromatic and N—H= 0.86 Å] and were refined using a riding-model approximation with *U*_iso_(H) constrained to 1.2 times *U*_eq_ of the respective parent atom.

### Crystal data

**Table:**

C_10_H_7_FN_2_OS	*D*_x_ = 1.534 Mg m^−^^3^
*M**_r_* = 222.24	Melting point: 443(1) K
Monoclinic, *P*2_1_/*c*	Mo *K*α radiation, λ = 0.71073 Å
*a* = 12.2171 (8) Å	Cell parameters from 8732 reflections
*b* = 5.0741 (3) Å	θ = 3.3–27.5°
*c* = 15.7078 (10) Å	µ = 0.32 mm^−^^1^
β = 98.820 (6)°	*T* = 295 K
*V* = 962.22 (11) Å^3^	Plate, colourless
*Z* = 4	0.40 × 0.17 × 0.08 mm
*F*(000) = 456	

### Data collection

**Table:**

Rigaku Pilatus 200K diffractometer	2169 independent reflections
Radiation source: Sealed tube_Mo	1556 reflections with *I* > 2σ(*I*)
Graphite Monochromator monochromator	*R*_int_ = 0.060
profile data from ω–scans	θ_max_ = 27.5°, θ_min_ = 3.3°
Absorption correction: multi-scan *CrystalClear*; Rigaku, 2008	*h* = −15→15
*T*_min_ = 0.701, *T*_max_ = 1.000	*k* = −6→6
8722 measured reflections	*l* = −20→20

### Refinement

**Table:**

Refinement on *F*^2^	Primary atom site location: structure-invariant direct methods
Least-squares matrix: full	Secondary atom site location: difference Fourier map
*R*[*F*^2^ > 2σ(*F*^2^)] = 0.046	Hydrogen site location: inferred from neighbouring sites
*wR*(*F*^2^) = 0.098	H-atom parameters constrained
*S* = 0.89	*w* = 1/[σ^2^(*F*_o_^2^) + (0.0481*P*)^2^] where *P* = (*F*_o_^2^ + 2*F*_c_^2^)/3
2169 reflections	(Δ/σ)_max_ < 0.001
136 parameters	Δρ_max_ = 0.22 e Å^−^^3^
0 restraints	Δρ_min_ = −0.30 e Å^−^^3^

### Special details

**Table:**

Geometry. All esds (except the esd in the dihedral angle between two l.s. planes) are estimated using the full covariance matrix. The cell esds are taken into account individually in the estimation of esds in distances, angles and torsion angles; correlations between esds in cell parameters are only used when they are defined by crystal symmetry. An approximate (isotropic) treatment of cell esds is used for estimating esds involving l.s. planes.
Refinement. Refinement of *F*^2^ against ALL reflections. The weighted *R*-factor *wR* and goodness of fit *S* are based on *F*^2^, conventional *R*-factors *R* are based on *F*, with *F* set to zero for negative *F*^2^. The threshold expression of *F*^2^ > σ(*F*^2^) is used only for calculating *R*-factors(gt) *etc*. and is not relevant to the choice of reflections for refinement. *R*-factors based on *F*^2^ are statistically about twice as large as those based on *F*, and *R*- factors based on ALL data will be even larger.

### Fractional atomic coordinates and isotropic or equivalent isotropic displacement parameters (Å^2^)

**Table:**

	*x*	*y*	*z*	*U*_iso_*/*U*_eq_	
S1	0.18106 (4)	0.17354 (10)	0.85154 (3)	0.04570 (16)	
F1	0.21170 (8)	0.5331 (2)	1.16166 (6)	0.0518 (3)	
C1	0.30360 (13)	0.7730 (4)	1.06395 (10)	0.0358 (4)	
O1	0.31999 (9)	0.5432 (3)	0.93486 (8)	0.0496 (3)	
C8	0.11054 (13)	0.3206 (3)	0.92625 (10)	0.0343 (4)	
N1	0.15631 (11)	0.5105 (3)	0.98371 (9)	0.0383 (3)	
H1	0.1156	0.5758	1.0185	0.046*	
C3	0.32638 (15)	0.8821 (4)	1.21586 (12)	0.0479 (5)	
H3	0.3093	0.8492	1.2706	0.057*	
N2	0.01031 (11)	0.2339 (3)	0.92704 (9)	0.0398 (3)	
C2	0.28064 (13)	0.7317 (4)	1.14663 (11)	0.0380 (4)	
C5	0.42365 (16)	1.1283 (4)	1.12149 (14)	0.0533 (5)	
H5	0.4722	1.2635	1.1129	0.064*	
C9	−0.01271 (15)	0.0323 (4)	0.86792 (11)	0.0432 (4)	
H9	−0.0799	−0.0572	0.8604	0.052*	
C7	0.26161 (13)	0.6006 (4)	0.98871 (10)	0.0362 (4)	
C10	0.06810 (15)	−0.0259 (4)	0.82218 (11)	0.0466 (5)	
H10	0.0636	−0.1565	0.7802	0.056*	
C6	0.37735 (14)	0.9737 (4)	1.05305 (12)	0.0442 (4)	
H6	0.3959	1.0045	0.9987	0.053*	
C4	0.39778 (16)	1.0819 (4)	1.20273 (14)	0.0549 (5)	
H4	0.4289	1.1864	1.2488	0.066*	

### Atomic displacement parameters (Å^2^)

**Table:**

	*U*^11^	*U*^22^	*U*^33^	*U*^12^	*U*^13^	*U*^23^
S1	0.0485 (3)	0.0515 (3)	0.0392 (3)	0.0029 (2)	0.01349 (19)	−0.0103 (2)
F1	0.0561 (6)	0.0604 (8)	0.0405 (6)	−0.0113 (6)	0.0126 (5)	0.0059 (5)
C1	0.0343 (8)	0.0378 (9)	0.0358 (9)	0.0023 (8)	0.0067 (6)	−0.0007 (7)
O1	0.0485 (7)	0.0616 (9)	0.0425 (7)	−0.0039 (7)	0.0198 (6)	−0.0084 (6)
C8	0.0403 (9)	0.0355 (9)	0.0279 (8)	0.0032 (7)	0.0077 (6)	0.0002 (7)
N1	0.0380 (7)	0.0433 (9)	0.0351 (7)	−0.0017 (7)	0.0109 (6)	−0.0090 (6)
C3	0.0443 (9)	0.0626 (13)	0.0361 (9)	0.0066 (9)	0.0042 (7)	−0.0049 (9)
N2	0.0415 (8)	0.0404 (8)	0.0385 (8)	−0.0017 (7)	0.0097 (6)	−0.0049 (7)
C2	0.0345 (8)	0.0425 (10)	0.0375 (9)	0.0023 (8)	0.0065 (6)	0.0019 (8)
C5	0.0442 (10)	0.0454 (12)	0.0687 (14)	−0.0062 (9)	0.0037 (9)	−0.0016 (10)
C9	0.0482 (10)	0.0382 (10)	0.0421 (10)	−0.0024 (9)	0.0037 (8)	−0.0026 (8)
C7	0.0392 (8)	0.0370 (10)	0.0335 (9)	0.0012 (8)	0.0088 (7)	0.0014 (7)
C10	0.0588 (11)	0.0410 (11)	0.0392 (10)	0.0036 (9)	0.0047 (8)	−0.0094 (8)
C6	0.0401 (9)	0.0467 (11)	0.0470 (10)	−0.0022 (8)	0.0105 (8)	0.0036 (9)
C4	0.0489 (10)	0.0569 (13)	0.0551 (12)	0.0012 (10)	−0.0045 (9)	−0.0170 (11)

### Geometric parameters (Å, º)

**Table:**

S1—C10	1.716 (2)	C3—C2	1.375 (2)
S1—C8	1.7280 (16)	C3—H3	0.9300
F1—C2	1.357 (2)	N2—C9	1.381 (2)
C1—C2	1.386 (2)	C5—C6	1.380 (3)
C1—C6	1.388 (2)	C5—C4	1.381 (3)
C1—C7	1.496 (2)	C5—H5	0.9300
O1—C7	1.2223 (18)	C9—C10	1.340 (2)
C8—N2	1.303 (2)	C9—H9	0.9300
C8—N1	1.379 (2)	C10—H10	0.9300
N1—C7	1.356 (2)	C6—H6	0.9300
N1—H1	0.8600	C4—H4	0.9300
C3—C4	1.373 (3)		
			
C10—S1—C8	88.45 (8)	C6—C5—H5	120.1
C2—C1—C6	117.07 (16)	C4—C5—H5	120.1
C2—C1—C7	123.89 (16)	C10—C9—N2	115.65 (16)
C6—C1—C7	118.82 (15)	C10—C9—H9	122.2
N2—C8—N1	121.13 (14)	N2—C9—H9	122.2
N2—C8—S1	115.33 (13)	O1—C7—N1	121.90 (16)
N1—C8—S1	123.50 (12)	O1—C7—C1	121.34 (15)
C7—N1—C8	124.02 (14)	N1—C7—C1	116.75 (14)
C7—N1—H1	118.0	C9—C10—S1	110.75 (14)
C8—N1—H1	118.0	C9—C10—H10	124.6
C4—C3—C2	118.77 (18)	S1—C10—H10	124.6
C4—C3—H3	120.6	C5—C6—C1	121.16 (18)
C2—C3—H3	120.6	C5—C6—H6	119.4
C8—N2—C9	109.78 (14)	C1—C6—H6	119.4
F1—C2—C3	117.53 (15)	C3—C4—C5	120.35 (18)
F1—C2—C1	119.71 (15)	C3—C4—H4	119.8
C3—C2—C1	122.75 (17)	C5—C4—H4	119.8
C6—C5—C4	119.88 (19)		
			
C10—S1—C8—N2	−1.98 (14)	C8—N1—C7—O1	9.4 (3)
C10—S1—C8—N1	175.93 (15)	C8—N1—C7—C1	−170.15 (15)
N2—C8—N1—C7	177.02 (16)	C2—C1—C7—O1	−140.34 (18)
S1—C8—N1—C7	−0.8 (2)	C6—C1—C7—O1	34.1 (3)
N1—C8—N2—C9	−175.59 (15)	C2—C1—C7—N1	39.2 (2)
S1—C8—N2—C9	2.38 (19)	C6—C1—C7—N1	−146.37 (16)
C4—C3—C2—F1	179.02 (16)	N2—C9—C10—S1	0.2 (2)
C4—C3—C2—C1	0.0 (3)	C8—S1—C10—C9	0.94 (14)
C6—C1—C2—F1	−178.05 (14)	C4—C5—C6—C1	0.8 (3)
C7—C1—C2—F1	−3.6 (3)	C2—C1—C6—C5	−1.3 (3)
C6—C1—C2—C3	0.9 (3)	C7—C1—C6—C5	−176.13 (16)
C7—C1—C2—C3	175.41 (16)	C2—C3—C4—C5	−0.6 (3)
C8—N2—C9—C10	−1.6 (2)	C6—C5—C4—C3	0.2 (3)

### Hydrogen-bond geometry (Å, º)

**Table:**

*D*—H···*A*	*D*—H	H···*A*	*D*···*A*	*D*—H···*A*
N1—H1···N2^i^	0.86	2.11	2.944 (2)	165
C3—H3···O1^ii^	0.93	2.62	3.474 (2)	153

Symmetry codes: (i) −*x*, −*y*+1, −*z*+2; (ii) *x*, −*y*+3/2, *z*+1/2.
