# Supplementary figures and images for: Crystal structure of 2-fluoro-N-(1,3-thia­zol-2-yl)benzamide
Source: Acta Crystallogr E Crystallogr Commun. 2015 Oct 24;71(Pt 11):o882–3. doi: 10.1107/S2056989015019192 (PMC4645012; doi:10.1107/S2056989015019192)

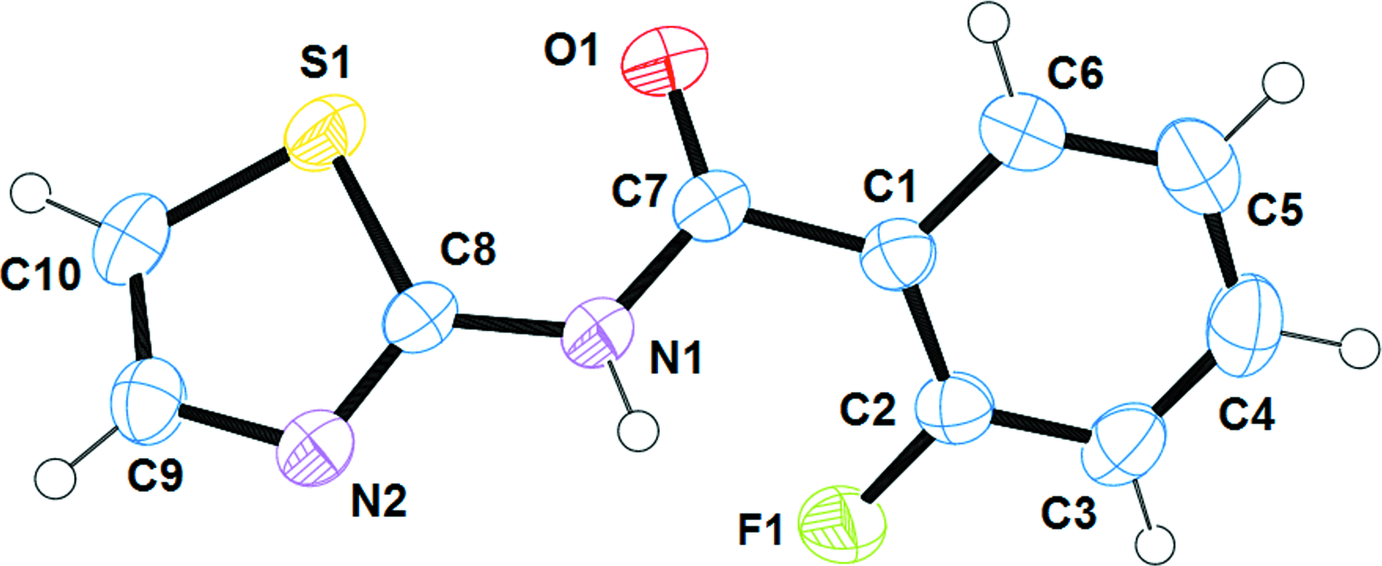

Supplement: Supplementary file 4 [file e-71-0o882-fig1.tif]

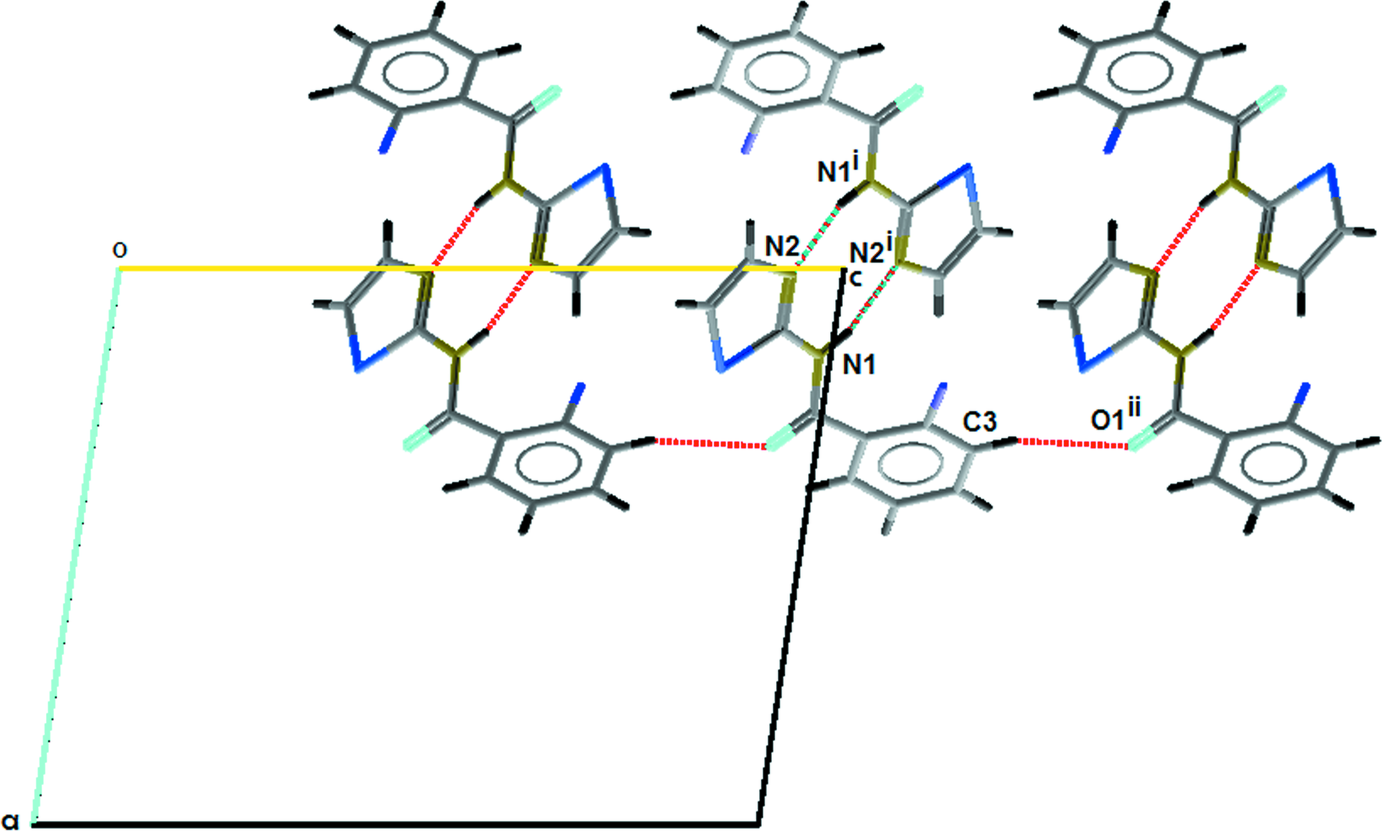

Supplement: Supplementary file 5 [file e-71-0o882-fig2.tif]
